# Diffuse Intraductal Breast Papillomatosis in a 34‐Year‐Old Female With Maffucci Syndrome: A Case Report

**DOI:** 10.1155/crra/7030550

**Published:** 2026-06-22

**Authors:** Eren Sakarcan, Jeanette Fulton

**Affiliations:** ^1^ University of South Carolina School of Medicine, Columbia, South Carolina, USA, musc.edu; ^2^ Department of Radiology, Prisma Health Midlands, Columbia, South Carolina, USA

**Keywords:** enchondromatosis, IDH mutation, intraductal papilloma, Maffucci syndrome, papillary breast lesion

## Abstract

**Purpose:**

This report aims to raise clinical awareness of a rare case of Maffucci syndrome in a 34‐year‐old female presenting with diffuse intraductal breast papillomatosis. It further explores a possible hypothesis‐generating pathogenic association between Maffucci syndrome and a specific subtype of papillary breast neoplasia in the context of shared molecular pathways involved in disease pathogenesis.

**Introduction:**

Maffucci syndrome is a rare, congenital, nonhereditary disorder characterized by enchondromas, hemangiomas, and skeletal deformities, typically presenting in early childhood. Since its initial description in 1881, fewer than 300 cases have been reported, with an estimated prevalence of < 1 in 27 million. Somatic mutations in IDH1 and IDH2 are key drivers of Maffucci syndrome and are also implicated in malignancies such as gliomas, chondrosarcomas, intrahepatic cholangiocarcinoma, and acute myeloid leukemia. Most relevant to this case, tall cell carcinoma with reversed polarity (TCCRP)—a rare subtype of papillary breast carcinoma—is characteristically associated with hotspot IDH2 R172 mutations, a molecular feature otherwise uncommon in both breast carcinomas and Maffucci syndrome. Only one documented case of an IDH1‐mutated solid papillary carcinoma with reversed polarity (SPCRP) exists in the current literature.

**Case Description:**

The patient is a 34‐year‐old female with Maffucci syndrome, diagnosed in childhood via clinical and radiographic evaluation, with a history of skeletal and vascular complications, including primary chondrosarcomas of the left scapula, right distal patella, and right proximal tibia. She initially presented in 2016 with left‐sided hemorrhagic nipple discharge, prompting serial imaging and biopsies that identified recurrent intraductal papillary lesions, consistently benign on core needle biopsy and surgical excision. A right‐sided lesion was excised in 2022, followed by two left‐sided lesions resected in 2023 and 2024. Mammography in November 2024 revealed three nodular lesions in the left lateral breast, characterized as complex cystic masses, with core needle biopsy confirming benign intraductal papillomas without atypia. Although subsequent imaging demonstrated stability of these lesions on follow‐up ultrasound in June 2025, the longitudinal course illustrates a recurrent pattern of intraductal papillary lesion development over time.

**Conclusions and Importance:**

This case raises the possibility of an association between Maffucci syndrome and recurrent papillary breast lesions. In the absence of molecular confirmation, this relationship remains speculative and should be regarded as hypothesis generating, underscoring the need for further investigation with genetic and immunohistochemical correlation rather than changes to established breast cancer screening guidelines.

## 1. Introduction

Maffucci syndrome is an exceedingly rare congenital, nonhereditary disorder characterized by enchondromas, hemangiomas, and skeletal deformities, typically presenting in early childhood [1]. Lymphangiomas are a rare additional feature [2]. Enchondromas primarily affect the hands and feet and carry a  > 30% risk of malignant transformation, most commonly to chondrosarcomas, gliomas, ovarian tumors, and other sarcomas [3, 4].

Since its initial description in 1881, fewer than 300 cases have been reported worldwide, with an estimated prevalence of < 1 in 27 million. A 2011 study identified sporadic heterozygous mutations in Isocitrate Dehydrogenase 1 and 2 (IDH1 and IDH2) as key drivers of Maffucci syndrome pathogenesis, with IDH1 variants being most prevalent [4]. IDH mutations also underlie Ollier disease (Type I enchondromatosis) and are implicated in multiple malignancies, including gliomas, chondrosarcomas, intrahepatic cholangiocarcinoma, acute myeloid leukemia, and astrocytomas [5]. However, IDH1 mutations account for only ~3% of all human cancers [6, 7].

Of particular relevance to this case, tall cell carcinoma with reversed polarity (TCCRP), also known as solid papillary carcinoma with reversed polarity (SPCRP), is a rare subtype of invasive papillary breast carcinoma characteristically associated with hotspot IDH2 R172 mutations [8, 9]. This represents a genetic link otherwise uncommon in both breast carcinomas and Maffucci syndrome [4, 10]. To date, only one case of an IDH1‐mutated TCCRP/SPCRP has been documented in the literature [9]. Further, to our knowledge, recurrent and/or diffuse intraductal papillomatosis in Maffucci syndrome has not been previously described.

## 2. Case Description

**Figure 1:**
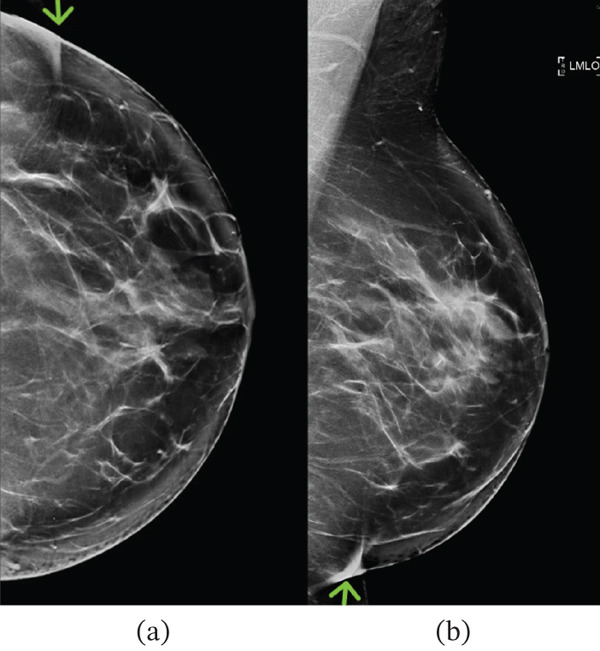
2D C‐view mammogram of the left breast in (a) CC and (b) MLO projections demonstrates postsurgical changes (green arrows) from prior reduction mammoplasty without discrete lesions in the breast or subareolar region. BI‐RADS 2 (benign).

**Figure 2:**
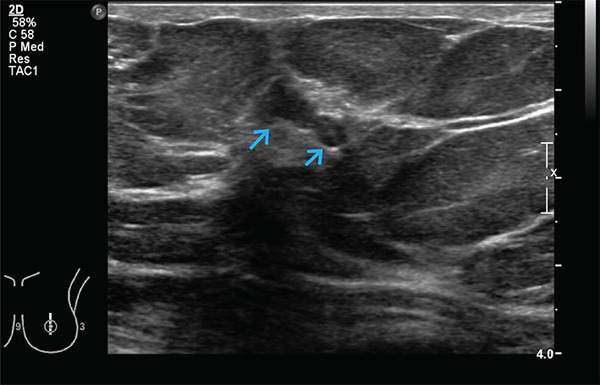
Targeted ultrasound of the subareolar left breast demonstrates a 12‐mm solid, oval, lobulated intraductal mass (blue arrows). BI‐RADS 4 (suspicious; biopsy recommended).

The patient is a 34‐year‐old female with a history of Maffucci syndrome, diagnosed in childhood via radiographic and clinical evaluation, and an oncologic history of chondrosarcomas of the left scapula, right distal patella, and right proximal tibia, as well as multiple hemangiomas of the left ankle, dorsum of the foot, and liver. She has no known family history of breast cancer or hereditary cancer syndromes, nor any psychosocial factors that would increase her risk of breast disease. She initially presented in April 2016 with single‐duct spontaneous bloody discharge from her left breast. A diagnostic mammogram was obtained and demonstrated architectural distortion from prior reduction mammoplasty without any discrete lesions in the breast or subareolar region, BI‐RADS 2 (benign) (Figure 1). Targeted ultrasound revealed a lobulated intraductal solid mass, BI‐RADS 4 (suspicious; biopsy recommended) (Figure 2). Ultrasound‐guided core needle biopsy demonstrated a papillary lesion without atypia, which was confirmed on surgical excision (histopathology). No immunostaining was performed.

**Figure 3:**
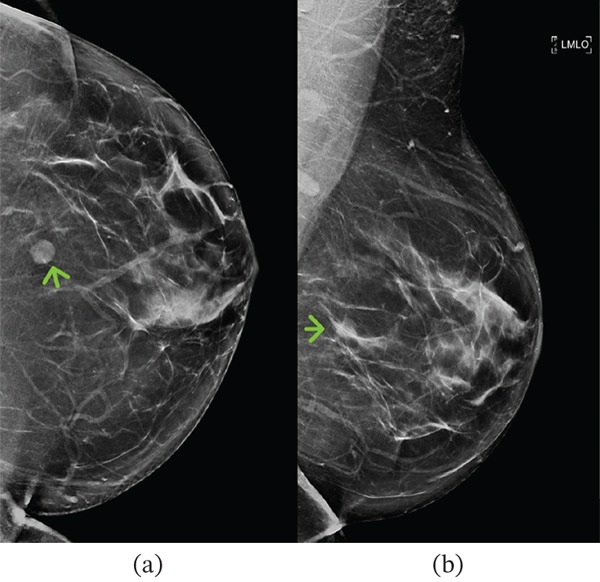
3D tomosynthesis mammogram of the left breast in (a) CC and (b) MLO projections demonstrates a circumscribed, round, 9‐mm mass (green arrow) deep in the breast, slightly lateral to the posterior nipple line. BI‐RADS 0 (incomplete; ultrasound recommended).

**Figure 4:**
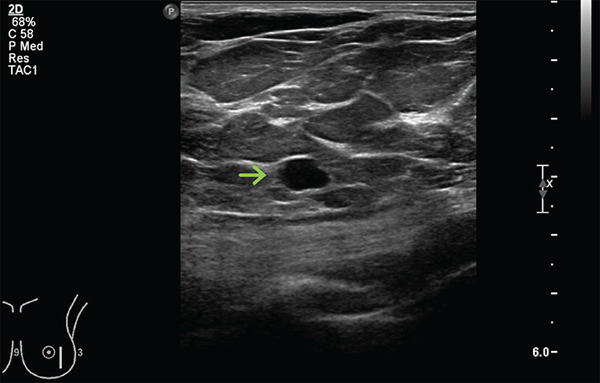
Ultrasound of the left breast in antiradial projection demonstrates a 9‐mm circumscribed, anechoic simple cyst with posterior acoustic enhancement at the 4 o′clock position (green arrow), corresponding to the mammographic finding in Figure 3. BI‐RADS 2 (benign).

The patient returned for mammography 5 years later in November 2021 for evaluation of a deep left breast nodule identified on routine chest CT surveillance for her chondrosarcoma. She had remained asymptomatic and had not undergone routine mammography since her 2016 surgical excision. Diagnostic mammogram revealed a posterior mass in her left breast (Figure 3), which was confirmed by ultrasound to be a circumscribed anechoic simple cyst, BI‐RADS 2 (benign) (Figure 4).

**Figure 5:**
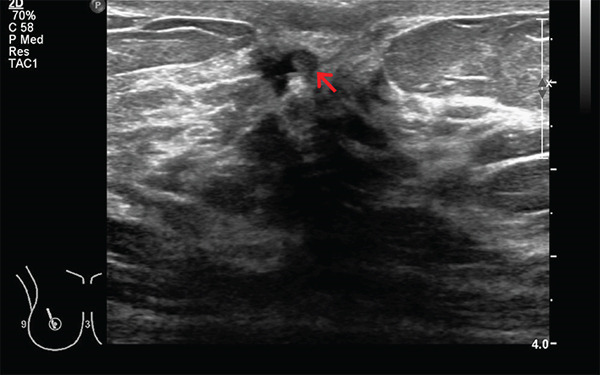
Ultrasound of the right subareolar breast in antiradial projection demonstrates a 3‐mm intraductal, solid mass at the 1 o′clock position (red arrow). BI‐RADS 4 (suspicious; biopsy recommended).

Ten months later, in October 2022, the patient presented with spontaneous clear single‐duct discharge from her right nipple. Mammography (not shown) was stable from prior exam. Ultrasound revealed a small, circumscribed solid mass in a retroareolar duct of the right breast, BI‐RADS 4 (suspicious; biopsy recommended) (Figure 5). Ultrasound‐guided core needle biopsy without immunostaining revealed a papillary lesion, which was surgically excised and found to be an intraductal papilloma with sclerosis, duct ectasia, usual ductal hyperplasia, and stromal fibrosis lacking atypia.

**Figure 6:**
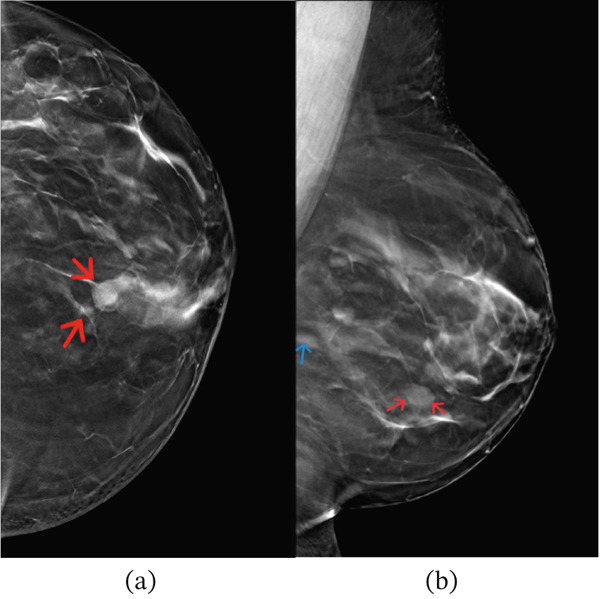
3D tomosynthesis mammogram of the left breast in (a) CC and (b) MLO projections demonstrates a new 11‐mm oval, circumscribed mass at the 6 o′clock position in the anterior breast (red arrows) and a stable mass at the 3 o′clock position in the posterior breast (blue arrows). BI‐RADS 4 (suspicious; biopsy recommended).

**Figure 7:**
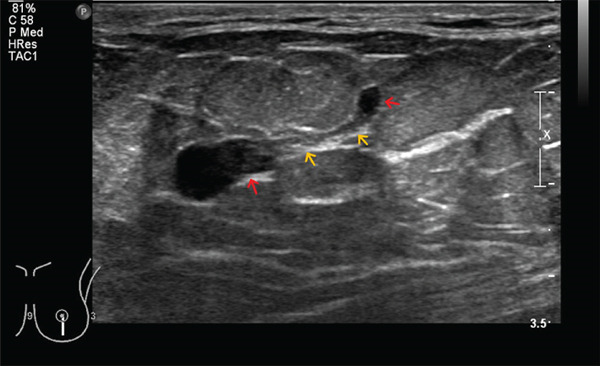
Ultrasound of the left breast at the 6 o′clock position in radial projection demonstrates two adjacent complex cystic and solid masses (red arrows), with intraductal solid tissue extending between them (yellow arrows), corresponding to the anterior mammographic findings in Figure 6. BI‐RADS 4 (suspicious; biopsy recommended).

One year later, in October 2023, follow‐up diagnostic mammography demonstrated a new 11‐mm oval, circumscribed mass in the anterior left breast and a stable lesion in the posterior retroareolar region, BI‐RADS 4 (suspicious; biopsy recommended) (Figure 6). Targeted ultrasound demonstrated two adjacent complex cystic and solid masses, consistent with papillary lesions, with an intraductal component extending between them (Figure 7). Ultrasound‐guided core needle biopsy revealed an atypical intraductal papillary lesion. However, subsequent surgical excision in January 2024 revealed an intraductal papilloma with usual ductal hyperplasia, columnar cell change, and microcalcifications, without atypia. Immunohistochemical staining showed patchy CK5/6 positivity and heterogeneous ER expression. These findings likely reflect the known intralesional heterogeneity of papillary breast lesions and sampling limitations of core needle biopsy.

**Figure 8:**
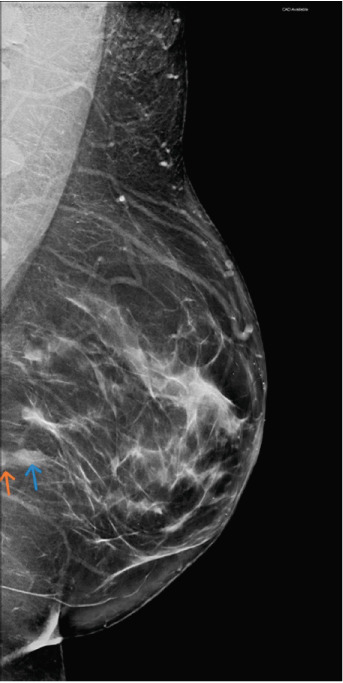
3D tomosynthesis slice of the left breast in MLO projection demonstrates enlargement of the 3 o′clock mass previously noted in 2023, now measuring 8 mm (blue arrow), and a new partially visualized mass at 4 o′clock (orange arrow). BI‐RADS 4 (suspicious; biopsy recommended).

**Figure 9:**
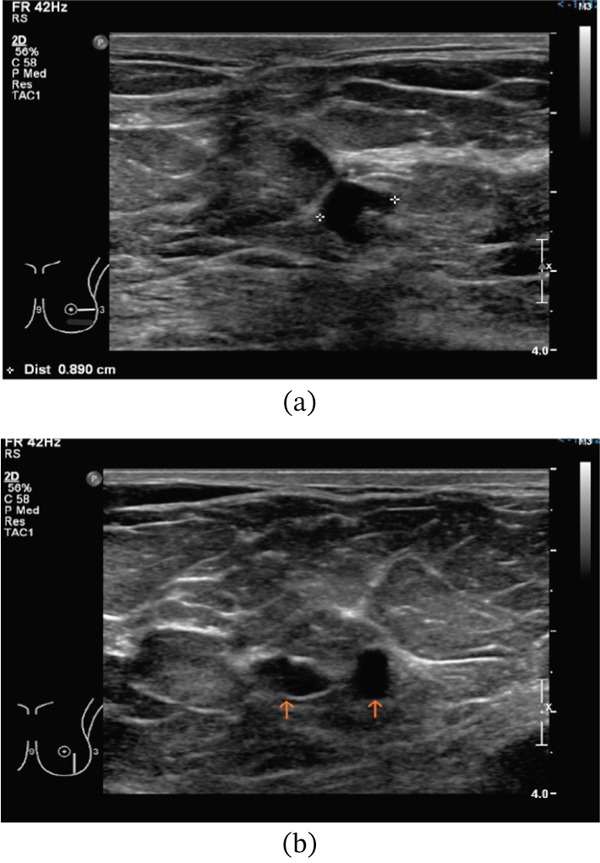
Ultrasound of the lateral left breast in (a) radial and (b) antiradial projections demonstrates (a) an approximately 9‐mm complex cystic/solid mass at the 3 o′clock position (white asterisks) and (b) two adjacent solid masses at the 4‐5 o′clock positions, measuring approximately 9 mm and 6 mm (orange arrows). BI‐RADS 4 (suspicious; biopsy recommended).

One year later, in November 2024, routine diagnostic mammography demonstrated enlargement of the posterior mass previously noted in October 2023, as well as a new mass in the posterior left breast, BI‐RADS 4 (suspicious; biopsy recommended) (Figure 8). Ultrasound (Figure 9) demonstrated a round, complex cystic/solid mass (a) at the 3 o’clock position and (b) two additional solid masses at the 4‐5 o’clock positions corresponding to the posterior mass partially visualized on the MLO projection (Figure 8, orange arrow). Three new complex cystic/solid lesions were also identified in the lower outer quadrant of her left breast, BI‐RADS 4 (suspicious; biopsy recommended). Ultrasound‐guided core needle biopsy revealed papillary lesions without atypia. Surgical excision was not performed following core needle biopsy.

**Figure 10:**
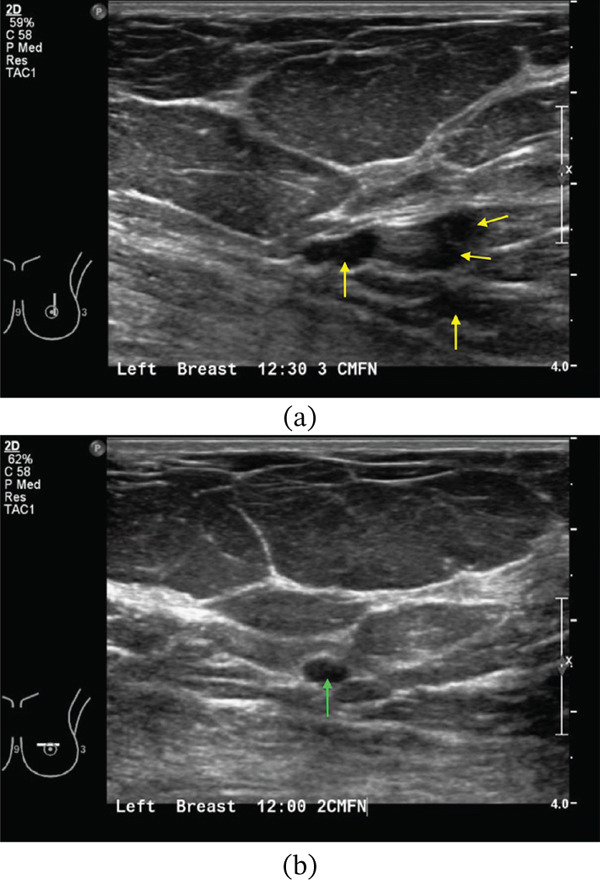
Ultrasound of the subareolar left breast in (a) radial and (b) antiradial projections demonstrates multiple complex cystic/solid masses at the 12 o′clock position ranging from 4 to 8 mm (yellow and green arrows). BI‐RADS 3 (probably benign).

In June 2025, left breast ultrasound (not shown) demonstrated stability of the lesions identified in November 2024. Follow‐up diagnostic mammography in November 2025 (not shown) demonstrated multiple small, circumscribed masses with smooth margins in the left breast, several of which were stable compared with 2024 findings. Targeted ultrasound revealed corresponding cystic lesions ranging from anechoic to hypoechoic and measuring 4–8 mm, BI‐RADS 3 (probably benign) (Figure 10). Short‐interval imaging follow‐up was recommended to confirm stability. The patient has a left breast diagnostic mammogram scheduled for May 2026. A timeline of the patient′s clinical course and associated imaging can be seen in Figure S1.

## 3. Discussion

This case contributes to the limited literature describing breast neoplasia in patients with Maffucci syndrome by illustrating a diffuse, recurrent pattern of intraductal papillary lesions involving both breasts over an extended follow‐up period. While isolated benign breast tumors have been reported in patients with Maffucci syndrome, including phyllodes tumors [11] and tubular adenomas [12], reports of recurrent or diffuse papillary breast disease in this population are exceedingly sparse. In this case, there was discordance between atypia suggested on core needle biopsy and absence of atypia on excision, likely reflecting known sampling limitations and intralesional heterogeneity of papillary breast lesions. The longitudinal nature of this case, the development of additional lesions over time, the bilateral distribution of lesions, and discussion of a possible IDH‐related mechanistic link together represent the primary added value of this report to the current literature.

From a surgical oncology standpoint, papillary breast lesions present challenges due to their heterogeneity and variable malignant potential [13]. Although many papillary lesions are benign, those exhibiting atypia or complex architecture may undergo malignant transformation or harbor foci of ductal carcinoma in situ (DCIS) not sampled by core needle biopsy. Furthermore, the unique link between IDH1/2 mutations and TCCRP/SPCRP raises the question of whether IDH‐mutant syndromes such as Maffucci syndrome could be associated with papillary breast neoplasia. In the absence of molecular testing of the breast lesions in this patient, this observation should be interpreted as hypothesis generating rather than evidence of a causal relationship and does not support changes to the current breast cancer screening guidelines [9].

In the general population, the decision to excise benign‐appearing papillary lesions is controversial, especially when core biopsy reveals no atypia [14]. A 2021 study attempted to establish more evidence‐based, precise criteria for surgical excision of such lesions, using malignancy upstage rate, atypia upstage rate, and lesion size. The study found that the upgrade rate (7.3%, 11/150) was greater for lesions ≥ 10 mm than for lesions < 10 mm (0.6%, 3/462; *p* = 0.01) [15].

These findings, together with the emergence of additional lesions in this patient over a relatively short span (8 years) and the presence of a known systemic tumor predisposition syndrome, may support consideration of closer imaging surveillance and improved discrimination between benign and potentially malignant papillary breast lesions in select patients. While current breast cancer screening guidelines do not account for Maffucci syndrome [16], this case raises the possibility that earlier or more frequent breast imaging may be beneficial in select cases. Tissue sampling of suspicious lesions should be considered, given the risk of malignancy upstaging, particularly for lesions ≥ 10 mm; however, we acknowledge the limited level of evidence inherent to this single case report.

## 4. Conclusion and Importance

We propose a potential IDH1‐/IDH2‐driven association between Maffucci syndrome and the development of recurrent papillary breast neoplasms. While this relationship remains unproven in the absence of molecular confirmation, the diffuse, recurrent, and bilateral distribution of papillary breast lesions observed in this patient over time, together with the known heterogeneity and malignant potential of papillary breast lesions, raises the possibility of an underlying IDH‐related mechanism and also suggests that individualized follow‐up strategies may warrant consideration in patients with tumor predisposition syndromes. These observations should be regarded as hypothesis generating and underscore the need for further investigation with molecular correlation, rather than changes to established breast cancer screening guidelines.

## Funding

No funding was received for this manuscript.

## Disclosure

An earlier version of this work was presented as an abstract at Discover USC, University of South Carolina, Columbia, South Carolina, April 21, 2023 [17].

## Ethics Statement

Ethical approval was not required for this case report in accordance with institutional policies, as it describes a single patient and does not constitute human subjects research. Written informed consent was obtained from the patient for publication of this case report and accompanying images. A copy of the written consent is available for review by the Editor upon request.

## Conflicts of Interest

The authors declare no conflicts of interest.

## Supporting information


**Supporting Information** Additional supplemental materials with supporting information may be found in Figure S1: The patient′s presentation and clinical course timeline of events with associated imaging and key findings as described in the Case Presentation.

## Data Availability

Data sharing is not applicable to this article as no datasets were generated or analyzed during the current study.

## References

[1] Mulliken J. B. , Maffucci Syndrome, National Organization for Rare Disorders, Published October 10, 2023, Accessed December 17, 2024. https://rarediseases.org/rare-diseases/maffucci-syndrome/.

[2] Jain A. , Cassuto J. , Sfakianaki E. , Kuker R. A. , Alizai H. , and Mohiuddin S. , The Utility of PET/CT for the Diagnosis of Periosteal Chondrosarcoma in a Patient With Maffucci′s Syndrome, Cureus. (2023) 15, no. 10, e46552, 10.7759/cureus.46552, 37822693. 37822693 PMC10563855

[3] McGarry M. E. , Long Term Oncologic Surveillance in Maffucci Syndrome: A Case Report, Journal of Oncological Sciences. (2017) 3, no. 3, 140–144, 10.1016/j.jons.2017.08.003.

[4] Pansuriya T. C. , van Eijk R. , d′Adamo P. , van Ruler M. A. J. H. , Kuijjer M. L. , Oosting J. , Cleton-Jansen A. M. , van Oosterwijk J. G. , Verbeke S. L. J. , Meijer D. , van Wezel T. , Nord K. H. , Sangiorgi L. , Toker B. , Liegl-Atzwanger B. , San-Julian M. , Sciot R. , Limaye N. , Kindblom L. G. , Daugaard S. , Godfraind C. , Boon L. M. , Vikkula M. , Kurek K. C. , Szuhai K. , French P. J. , and Bovée J. V. M. G. , Somatic Mosaic IDH1 and IDH2 Mutations Are Associated With Enchondroma and Spindle Cell Hemangioma in Ollier Disease and Maffucci Syndrome, Nature Genetics. (2011) 43, no. 12, 1256–1261, 10.1038/ng.1004, 22057234. 22057234 PMC3427908

[5] Wang Y.-P. , Di W.-J. , Qin S.-L. , Yang S. , Wang Z. , Xu Y.-F. , and Han P.-F. , A Rare Presentation of Maffucci Syndrome: A Case Report and Literature Review, Experimental and Therapeutic Medicine.(2023) 26, no. 3, 10.3892/etm.2023.12134, 37602309. PMC10433447 37602309

[6] National Library of Medicine , IDH1 Gene: Isocitrate Dehydrogenase (NADP(+)) 1, 2016, National Library of Medicine, Published February 1, 2016. Accessed April 17, 2025. http://medlineplus.gov/genetics/gene/idh1.

[7] Murugan A. K. and Alzahrani A. S. , Isocitrate Dehydrogenase IDH1 and IDH2 Mutations in Human Cancer: Prognostic Implications for Gliomas, British Journal of Biomedical Science. (2022) 79, 10208, 10.3389/bjbs.2021.10208, 35996504. 35996504 PMC8915566

[8] Kulka J. , Madaras L. , Floris G. , and Lax S. F. , Papillary Lesions of the Breast, Virchows Archiv. (2022) 480, no. 1, 65–84, 10.1007/s00428-021-03182-7, 34734332. 34734332 PMC8983543

[9] Weisman P. , Yu Q. , Flynn C. , Rehrauer W. , and Xu J. , Solid-Papillary Carcinoma With Reverse Polarity (SPCRP) Harboring a Novel IDH1 R132C Mutation: A Case Confirming the Expected IDH1/IDH2 Dichotomy, Human Pathology: Case Reports. (2020) 21, no. 200396, 200396, 10.1016/j.ehpc.2020.200396.

[10] Amary M. F. , Damato S. , Halai D. , Eskandarpour M. , Berisha F. , Bonar F. , McCarthy S. , Fantin V. R. , Straley K. S. , Lobo S. , Aston W. , Green C. L. , Gale R. E. , Tirabosco R. , Futreal A. , Campbell P. , Presneau N. , and Flanagan A. M. , Ollier Disease and Maffucci Syndrome Are Caused by Somatic Mosaic Mutations of IDH1 and IDH2, Nature Genetics. (2011) 43, no. 12, 1262–1265, 10.1038/ng.994, 22057236. 22057236

[11] Fernández-Aguilar S. , Buxant F. , and Noël J. C. , Benign Phyllodes Tumor Associated With Maffucci′s Syndrome, Breast. (2004) 13, no. 3, 247–249, 10.1016/j.breast.2003.06.001, 15177431. 15177431

[12] Mazingi D. , Mbanje C. , Jakanani G. , Muguti G. I. , Mandizvidza V. , and Bopoto S. , Maffucci′s Syndrome in Association With Giant Tubular Adenoma of the Breast: Case Report and Literature Review, International Journal of Surgery Case Reports. (2019) 63, 147–152, 10.1016/j.ijscr.2019.09.012, 31585326. 31585326 PMC6796655

[13] Murray M. , Pathologic High-risk Lesions, Diagnosis and Management, Clinical Obstetrics & Gynecology. (2016) 59, no. 4, 727–732, 10.1097/grf.0000000000000234, 27681693. 27681693 PMC5079293

[14] Nayak A. , Carkaci S. , Gilcrease M. Z. , Liu P. , Middleton L. P. , Bassett R. L. , Zhang J. , Zhang H. , Coyne R. L. , Bevers T. B. , Sneige N. , and Huo L. , Benign Papillomas Without Atypia Diagnosed on Core Needle Biopsy: Experience From a Single Institution and Proposed Criteria for Excision, Clinical Breast Cancer. (2013) 13, no. 6, 439–449, 10.1016/j.clbc.2013.08.007, 24119786. 24119786 PMC4605914

[15] Lee S. , Wahab R. A. , Sobel L. , Zhang B. , Brown A. L. , Lewis K. , Vijapura C. , and Mahoney M. C. , Analysis of 612 Benign Papillomas Diagnosed at Core Biopsy: Rate of Upgrade to Malignancy, Factors Associated With Upgrade, and a Proposal for Selective Surgical Excision, American Journal of Roentgenology. (2021) 217, no. 6, 1299–1311, 10.2214/ajr.21.25832, 34008998. 34008998

[16] US Preventive Services Task Force , Nicholson W. K. , Silverstein M. , Wong J. B. , Barry M. J. , Chelmow D. , Coker T. R. , Davis E. M. , Jaén C. R. , Krousel-Wood M. , and Lee S. , Screening for Breast Cancer, Journal of the American Medical Association. (2024) 331, no. 22, 1918–1930, 10.1001/jama.2024.5534. 38687503

[17] Sakarcan E. and Fulton J. , Diffuse Intraductal Papillomatosis in a 34-Year-Old Female: A Rare Case Presentation, 2023, University of South Carolina, https://sc.edu/about/signature_events/discover_uofsc/find_presenter/?t=%26fmt=%26cat=%26sctn=%26plname=sakarcan%26pcamp=10%26pdept=%26req=%26lcomm=%26mlname=%26mcamp=%26mdept=%26subsearch=Search.

