# Supplementary material for: Diffuse Intraductal Breast Papillomatosis in a 34‐Year‐Old Female With Maffucci Syndrome: A Case Report
Source: Case Rep Radiol. 2026 Jun 22;2026:7030550. doi: 10.1155/crra/7030550 (PMC13284830; doi:10.1155/crra/7030550)
Supplement: Supplementary file 1 — Supporting Information Additional supplemental materials with supporting information may be found in Figure S1: The patient′s presentation and clinical course timeline of events with associated imaging and key findings as described in the Case Presentation. [file CRRA-2026-7030550-s001.pdf]

## APR. 2016

### The patient initially presented with **single duct left breast bloody discharge**

Mammogram demonstrated architectural distortion from prior reduction mammoplasty ***without* any discrete lesions noted within the breast or subareolar location, BI-RADS 2** (Figure 1)

Ultrasound revealed an **lobulated intraductal solid mass, BI-RADS 4** (Figure 2)

US-guided needle biopsy demonstrates a papillary lesion without atypia confirmed on surgical excision

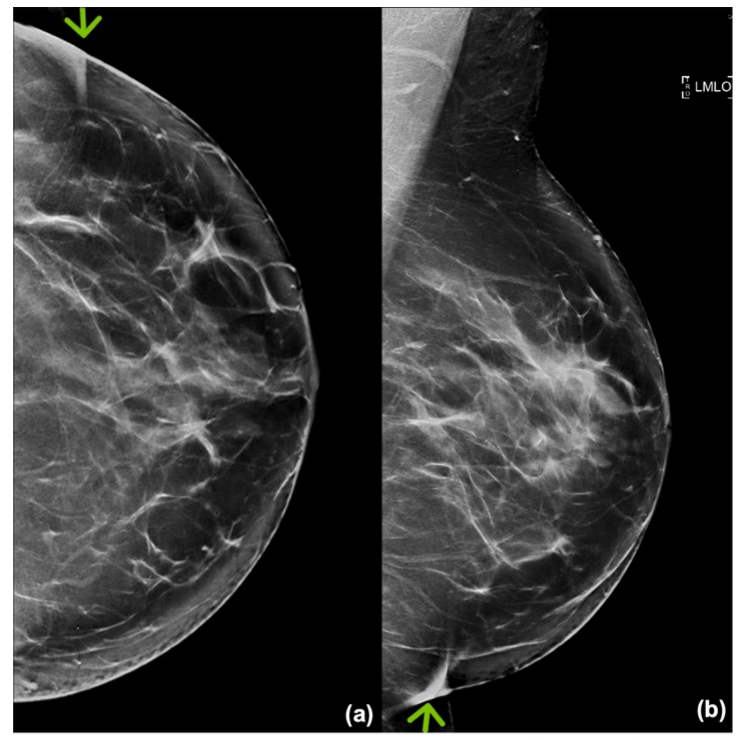

**Figure 1.** 2D C-view mammogram of the left breast in CC **(a)** and MLO **(b)** projections. Post-surgical changes **(green arrows)** from prior reduction mammoplasty

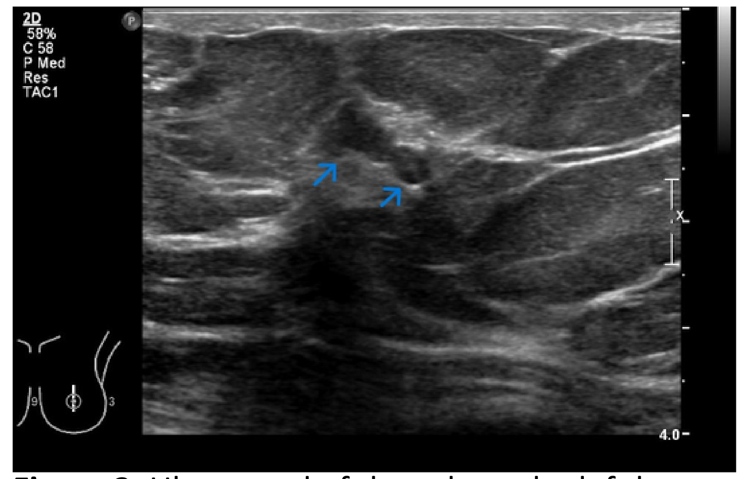

**Figure 2.** Ultrasound of the subareolar left breast demonstrates 12 mm solid, oval, lobulated intraductal mass **(blue arrows)**.

## NOV. 2021

### She **presented to mammography** for workup of a **deep left breast nodule** found on routine CT surveillance for her chondrosarcoma.

The patient had remained asymptomatic and did not have routine mammography following her 2016 surgical excision

Diagnostic mammography revealed a **mass** in the **posterior third of her left breast, BI-RADS 0 (incomplete, ultrasound recommended)** (Figure 3)

Ultrasound confirmed a **simple cyst, BI-RADS 2** (Figure 4)

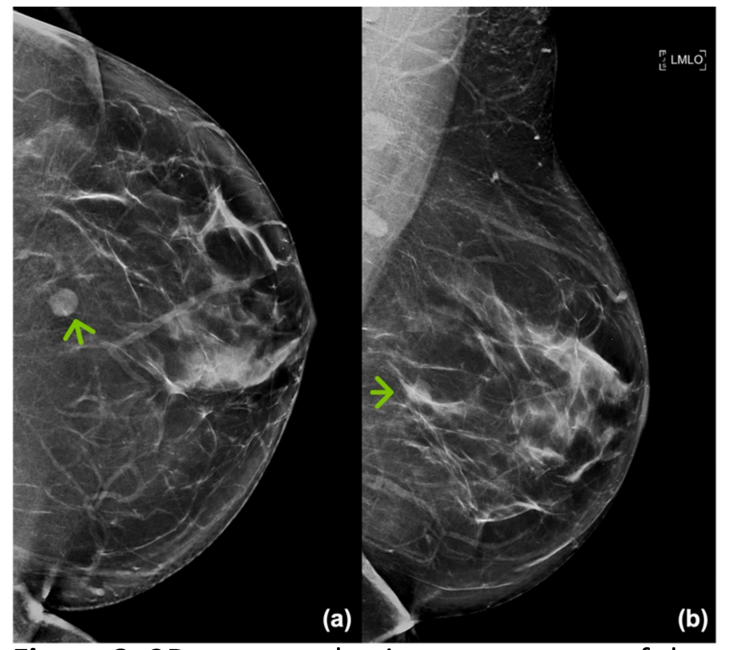

**Figure 3.** 3D tomosynthesis mammogram of the left breast in CC **(a)** and MLO **(b)** projections demonstrates a circumscribed, 9 mm nodule **(green arrow)** deep in the breast, slightly lateral to the posterior nipple line.

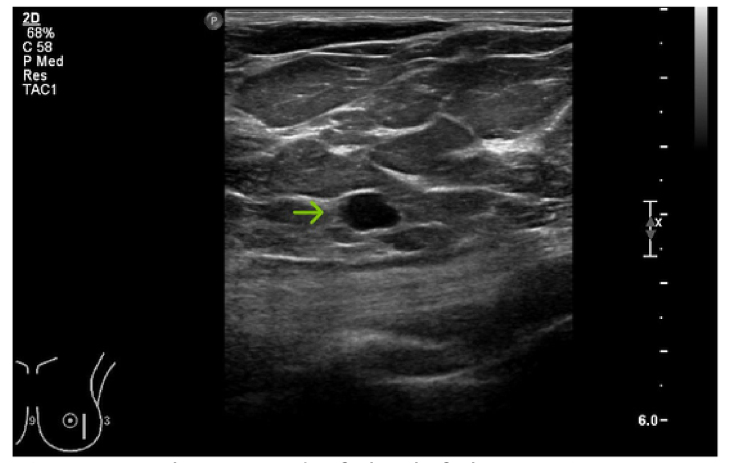

**Figure 4.** Ultrasound of the left breast in anti-radial projection demonstrates a 9 mm circumscribed, anechoic simple cyst with posterior acoustic enhancement at the 4 o'clock position **(green arrow)**, corresponding to the mammographic finding in Figure 3.

## OCT. 2022

### She returned 10 months later with **spontaneous clear single duct discharge** from the **right nipple**

Mammography (not shown) was **unremarkable)** except for architectural changes consistent with prior reduction mammoplasty

Ultrasound showed a **small, circumscribed solid mass in retroareolar duct on the right, BI-RADS 4** (Figure 5)

US-guided needle biopsy revealed a **papillary lesion**

Surgical excision confirmed the presence of a **papillary lesion *without* atypia**

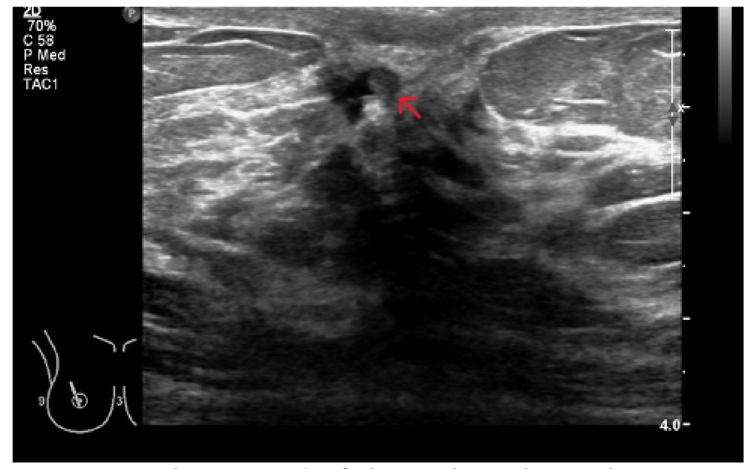

**Figure 5.** Ultrasound of the right subareolar breast in anti-radial projection demonstrates a 3 mm intraductal, solid mass at the 1 o'clock position **(red arrow)**.

## OCT. 2023

### Follow-up diagnostic mammography demonstrated **at least two new nodular densities** in the **left breast**

Diagnostic mammogram demonstrated **two new masses** in the **anterior left breast** and a **third lesion** in the **posterior left breast, BI-RADS 4** (Figure 6)

Ultrasound demonstrated **two complex cystic/solid masses** and an **intraductal component extended between them, BI-RADS 4** (Figure 7)

US-guided needle biopsy revealed an ***atypical* papillary lesion**

Surgical excision showed a **papillary lesion *without* atypia**

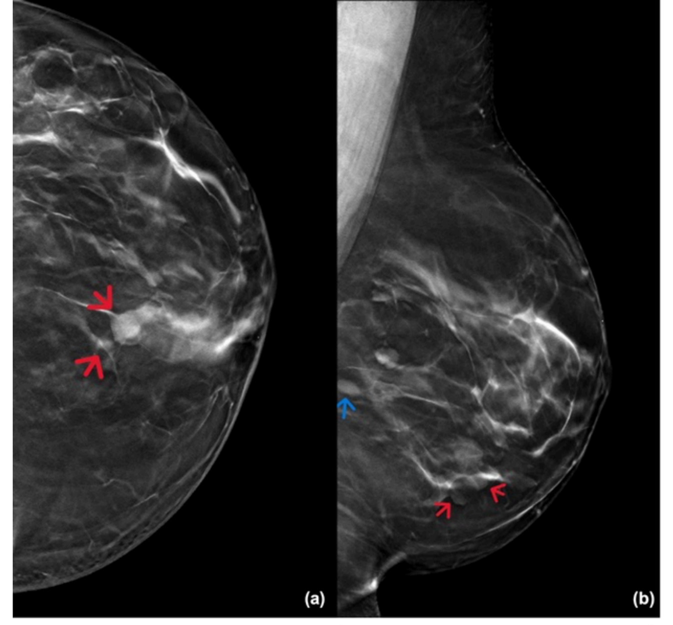

**Figure 6.** 3D tomosynthesis mammogram of the left breast in CC **(a)** and MLO **(b)** projections demonstrates a new 11 mm oval, circumscribed nodule at the 6 o'clock position in the anterior breast **(red arrows)** and a stable nodule at the 3 o'clock position in the posterior breast **(blue arrows)**.

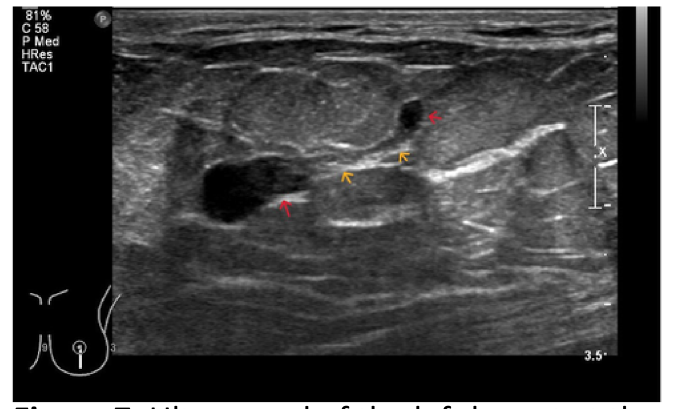

**Figure 7.** Ultrasound of the left breast at the 6 o'clock position in radial projection demonstrates two adjacent complex cystic and solid masses **(red arrows)**, with intraductal solid tissue extending between them **(yellow arrows)**, corresponding to the anterior mammographic findings in Figure 6.

## NOV. 2024

### She presented one year later for **routine diagnostic mammography** with **new masses** in the **left breast**

Mammography demonstrated **enlargement** of the **posterior mass previously noted in 2023**, and **two new masses in the left breast, BI-RADS 4** (Figure 8).

Ultrasound demonstrated a **round, hypoechoic, solid mass** and **two adjacent solid masses, BI-RADS 4** (Figure 9).

Core needle biopsy revealed **papillary lesions *without* atypia**

The patient **opted for follow-up in lieu of surgical excision**

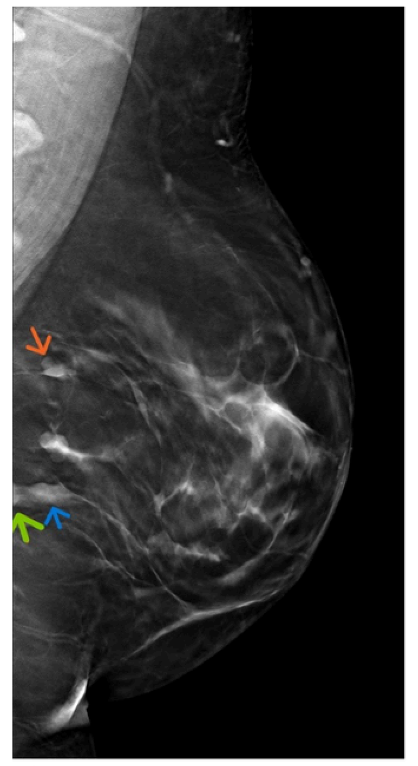

**Figure 8.** 3D tomosynthesis slice of the left breast in MLO projection demonstrates enlargement of the 3 o'clock mass previously noted in 2023, now measuring 8 mm **(blue arrow)**, and two new masses at the 4 o'clock (7 mm) and 5 o'clock (2 mm) positions of the posterior breast **(orange arrows)**.

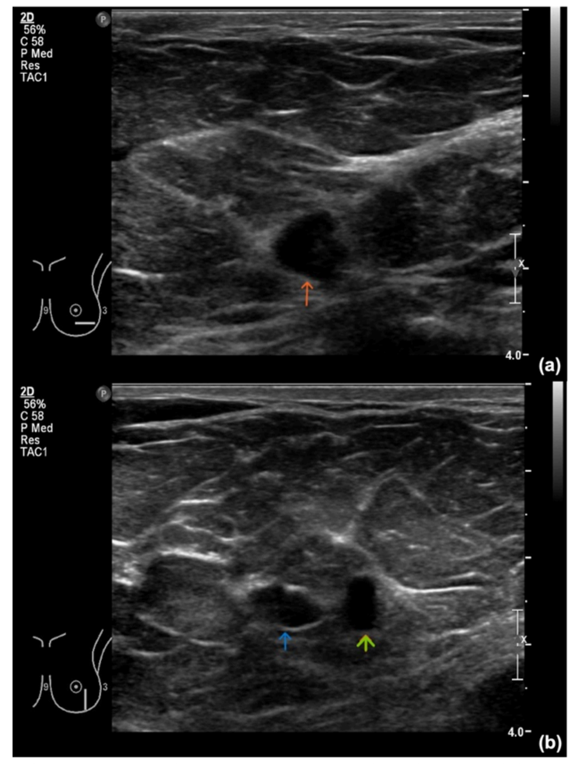

**Figure 9.** Ultrasound of the lateral left breast in anti-radial **(a)** and radial **(b)** projections demonstrates **(a)** an approximately 10 mm round, hypoechoic solid mass at the 4 o'clock position **(orange arrow)**, and **(b)** two adjacent solid masses at the 5 o'clock position measuring approximately 9 mm **(blue arrow)** and 6 mm **(green arrow)**.

## NOV. 2025

### She presented one year later for **routine diagnostic mammography** demonstrating **mostly stable findings with a few new small cystic lesions**

Mammography demonstrated **multiple small, circumscribed masses** with smooth margins in the left breast, **several of which were stable** compared with November 2024 findings

Ultrasound revealed corresponding **cystic lesions** ranging from anechoic to hypoechoic and measuring **4–8 mm, BI-RADS 3** (Figure 10)

**Short-interval imaging follow-up was recommended** to confirm stability

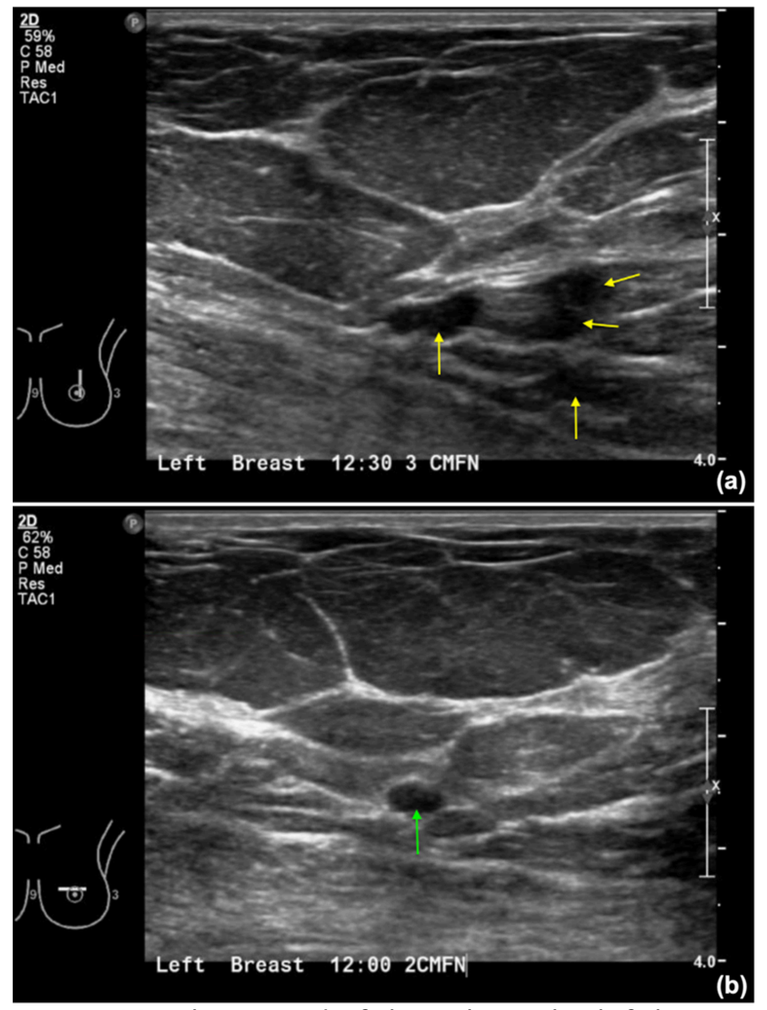


**Figure 10.** Ultrasound of the subareolar left breast in radial **(a)** and anti-radial **(b)** projections demonstrates multiple solid nodules with cystic features at the 6 o'clock position ranging from 4 mm to 8 mm **(yellow & green arrows)**.

## MAY 2026

### Follow-up **diagnostic mammogram** of the **left breast** is scheduled
